# Long-term clinical benefits of delayed-release cysteamine bitartrate capsules in patients with nephropathic cystinosis (response to “A comparison of immediate release and delayed release cysteamine in 17 patients with nephropathic cystinosis”)

**DOI:** 10.1186/s13023-023-02724-3

**Published:** 2023-06-23

**Authors:** Craig B. Langman

**Affiliations:** Division of Kidney Diseases, Department of Pediatrics, Feinberg School of Medicine, Northwestern University, The Ann and Robert H. Lurie Children’s Hospital of Chicago, Chicago, IL USA

**Keywords:** Nephropathic cystinosis, Delayed-release cysteamine, Immediate-release cysteamine, Cystine

## Abstract

The aim of this letter to the editor is to summarize the results from three clinical trial programs evaluating delayed-release cysteamine bitartrate (DR-CYS), which demonstrated the long-term clinical benefits in patients with nephropathic cystinosis when dosed every 12 h. The authors of “A comparison of immediate release and delayed release cysteamine in 17 patients with nephropathic cystinosis” presented recommendations altering the dosage and dosing scheme from what has been previously approved by the FDA for DR-CYS. In this letter to the editor, we critique the authors’ aforementioned article as it is a retrospective analysis of a small number of patients and does not follow the dosing recommendation by the FDA for equivalent dosing of DR-CYS to immediate-release cysteamine bitartrate (IR-CYS). In addition, the article does not include study data to properly support the authors’ suggestion of increased dosing effects and benefits. We present a summary of the results from the DR-CYS clinical trial program and evidence of the rigor from which the clinical data for DR-CYS were generated and recommendation for usage as prescribed.

**Supplementary Information:**

The online version contains supplementary material available at 10.1186/s13023-023-02724-3.

Dr Christina van Stein et al. recently published a retrospective analysis of 17 subjects with nephropathic cystinosis, assessing cysteamine plasma concentrations and mixed leukocyte cystine concentrations (white blood cell [WBC] cystine) after a single dose of immediate-release cysteamine bitartrate (IR-CYS) (Cystagon®, Mylan Pharmaceuticals, Canonsburg, PA and Recordati Pharma GmbH) as well as a single dose of delayed-release cysteamine bitartrate (DR-CYS) (Procysbi®; Horizon Therapeutics plc and Chiesi Farmaceutici S.p.A., Parma, Italy), respectively [1]. Data were collected during a three-year period and the dose of DR-CYS was reduced to 70% of the equivalent dose of IR-CYS. The investigators reported that IR- & DR-CYS effectively decreased WBC cystine below the therapeutic target, with fewer side effects occurring with DR-CYS. The authors also summarized their findings with a suggestion for increasing the dosing schedule of DR-CYS to three times daily to prevent a rapid decrease and achieve a steadier decline in cystine levels. It was also suggested that a more frequent dosing scheme of DR-CYS may ameliorate therapy adherence and improve the patients’ quality of life [1].

The clinical trial program for DR-CYS undeniably demonstrated the long-term clinical benefits of DR-CYS in nephropathic cystinosis when dosed every 12 h [2–5]. As principal investigator for these clinical trials, I want to reinforce the important findings from the clinical data demonstrating the efficacy and safety of DR-CYS when administered consistently every 12 h. All clinical data for DR-CYS were generated in prospective and controlled studies. The FDA-approval of DR-CYS resulted from an open-label, randomized, controlled, crossover trial (NCT01000961) of 43 subjects with cystinosis and was powered to show that DR-CYS administered every 12 h was non-inferior in the short-term (3-week) to IR-CYS for maintenance of WBC cystine at levels associated with optimal clinical outcomes [2]. The study met its primary endpoint, with the average steady-state total daily dose of DR-CYS being approximately 82% of the incoming steady-state total daily dose of IR-CYS [2]. Pharmacokinetic data showed therapeutic mean cysteamine plasma concentrations throughout the 12-hour dosing interval. No unexpected or serious safety concerns were experienced [2, 3].

Subsequently, we conducted a prospective, controlled, open label, single-arm extension trial (NCT 01197378) of DR-CYS for up to 5 years. Forty of the 43 subjects that participated in the first study enrolled in this extension trial. Throughout the first 24 months of study, mean WBC cystine was maintained at the therapeutic target and mean estimated glomerular filtration rate (eGFR) was also maintained. In children (≤ 18 years of age), linear growth velocity was maintained at 24 months compared with baseline height Z-scores. Also, the Pediatric Quality of Life Inventory measured significant improvements in social function, school function, and total function scores, which persisted for 24 months [3]. In this extension trial, 20 subjects continued treatment for longer than 60 months. During this extended treatment study with DR-CYS, mean WBC cystine was maintained at target levels and mean estimates of renal function as measured by eGFR were maintained. No unexpected or serious safety concerns were experienced [2, 3].

And finally, a long-term (up to 18 months) prospective, open-label evaluation (NCT01744782) of repeat twice-daily dosing of DR-CYS was performed to assess its safety and effectiveness in 15 cystinosis patients < 6 years of age who were naïve to any form of cysteamine treatment. This study also evaluated a new treatment initiation and titration methodology designed to maximize tolerability in treatment-naïve young children. There was a clinically meaningful decrease in mean WBC cystine levels over the treatment period, with 76.9% of patients reaching the treatment target by study exit and a significant improvement in eGFR, with a mean increase of 8.14 ± 15.48 ml/min/1.73 m^2^ at study exit [1, 5]. The investigators also observed clinically meaningful increases in mean Z score standing height and in mean Z score weight over the course of the treatment period. Overall, the pharmacokinetics in patients between the ages of 1 and 5 years of age was comparable with those in older children and adults [5].

The long-term clinical benefits of DR-CYS administered every 12 h have been demonstrated in a diverse group of patients with nephropathic cystinosis, ranging from 1 to 32 years of age. Consistent, long-term cysteamine-depleting therapy with DR-CYS has shown quantitative measurements of clinical benefits using biomarkers of disease control as well as measured improvements in pediatric quality of life, when studied prospectively in a controlled fashion. Pharmacokinetic data showed therapeutic plasma concentrations throughout the 12-hour dosing interval. There is no need to suggest using DR-CYS more than twice daily to effectively control nephropathic cystinosis. The burden of doing so clearly outweighs the potential benefits as our prospective controlled trials clearly demonstrated that twice daily dosing was optimal for control of this disease.

## Electronic supplementary material

Below is the link to the electronic supplementary material.

Supplementary Material 1

## Data Availability

Not applicable.

## List of Abbreviations

DR-CYS: Delayed-release cysteamine bitartrate
eGRF: Estimated glomerular filtration rate
IR-CYS: Immediate-release cysteamine bitartrate
WBC: White blood cell

## References

[1] van Stein C, Klank S, Grüneberg M, Ottolenghi C, Grebe J, Reunert J, Harms E, Marquardt T. 2021. A comparison of immediate release and delayed release cysteamine in 17 patients with nephropathic cystinosis. Orphanet J Rare Diseases, 16(1). 10.1186/s13023-021-01991-2 PMC8438894 34521447

[2] PROCYSBI [Prescribing Information], Dublin. Ireland: Horizon Pharma Ireland Ltd; 2020.

[3] Langman C, Greenbaum L, Sarwal M, Grimm P, Niaudet P, Deschênes G, Cornelissen E, Morin D, Cochat P, Matossian D, Gaillard S, Bagger M, Rioux P (2012). A randomized controlled crossover trial with delayed-release cysteamine bitartrate in nephropathic cystinosis: effectiveness on White Blood cell cystine levels and comparison of Safety. Clin J Am Soc Nephrol.

[4] Langman C, Greenbaum L, Grimm P, Sarwal M, Niaudet P, Deschenes G, Cornelissen E, Morin D, Cochat P, Elenberg E, Hanna C, Gaillard S, Bagger M, Rioux P (2014). Quality of life is improved and kidney function preserved in patients with nephropathic cystinosis treated for 2 years with delayed-release cysteamine bitartrate. J Pediatr.

[5] Vaisbich M, Caires Ferreira J, Price H, Young K, Sile S, Checani G, Langman C (2021). Cysteamine bitartrate delayed-release capsules control leukocyte cystine levels and promote statural growth and kidney health in an open‐label study of treatment‐naïve patients < 6 years of age with nephropathic cystinosis. JIMD Rep.

