# Supplementary material for: Long-term clinical benefits of delayed-release cysteamine bitartrate capsules in patients with nephropathic cystinosis (response to “A comparison of immediate release and delayed release cysteamine in 17 patients with nephropathic cystinosis”)
Source: Orphanet J Rare Dis. 2023 Jun 23;18:162. doi: 10.1186/s13023-023-02724-3 (PMC10290342; doi:10.1186/s13023-023-02724-3)
Supplement: Supplementary file 1 — Supplementary Material 1 [file 13023_2023_2724_MOESM1_ESM.docx]

OJRD-D-22-00127

Long-term Clinical Benefits of Delayed-release Cysteamine Bitartrate Capsules in Patients with Nephropathic Cystinosis (Response to “A comparison of immediate release and delayed release cysteamine in 17 patients with nephropathic cystinosis”)
Craig B Langman
Orphanet Journal of Rare Diseases

Dear Dr. Corrochano,

Thank you for the thorough assessment of the letter 'Long-term Clinical Benefits of Delayed-release Cysteamine Bitartrate Capsules in Patients with Nephropathic Cystinosis (Response to “A comparison of immediate release and delayed release cysteamine in 17 patients with nephropathic cystinosis”)' (OJRD-D-22-00127).

A point-by-point response along with “tracked changes” and “clean” revised documents have been resubmitted.

Reviewer reports:

Reviewer #1: Dear Prof Langman

Your letter provides a nice summary of the seminal prospective trials which lead to FDA approval and dosing of delayed-release cysteamine bitartrate in patients with cystinosis. You described those trials design and results, with special mention of delayed-release cysteamine bitartrate pharmacokinetic data, and therapeutic plasma concentrations achieved throughout the 12-hour dosing interval.

This letter content is rigorous and remarks the fact that retrospective observations from small group of patients are not comparable with controlled prospective randomized and open label clinical trials, in order to avoid potential bias in the field of rare diseases.

**Author Response:**

**Thank you for your assessment. We greatly appreciate the time you dedicated to reviewing this letter.**

Reviewer #2: We agree with the author (C. Langman) of this Letter to the Editor that, as demonstrated in the study by Langman et al. (CJASN, 2012), treatment with DR-CYS was non-inferior to IR-CYS, in terms of the fact that the difference observed in the mean peak value of WBC cystine level at 12 hours following administration with DR-CYS was not statistically inferior in comparison to the mean peak WBC cystine value 6 hours following administration with IR-CYS, and that the mean WBC cystine values are below the WBC cystine level target values (< 1 nmol ½ cystine/mg protein) associated with optimal clinical outcomes.
In contrast, the authors of the study described by Van Stein et al. (Orphanet Journal of Rare Diseases, 2021) argue that, based on their observation that following administration of DR-CYS, average WBC cystine levels reach a level above 0.5 nmol cystine/mg protein already after 250min, which is 230min or about 4 hours prior to the next dose of DR-CYS, the dosing interval of DR-CYS should be shortened, and thus dosing frequency of DR-CYS should be increased from twice-daily to three times daily. However, this observation by Stein C. et al is indeed the only basis for changing the recommendation on the dosing frequency of DR-CYS. Indeed, in the 24-months follow-up study by Langman et al. (Journal of Pediatrics, 2014), it was robustly demonstrated that during the entire duration of the study, the average WBC cystine levels remained below the level of 1 nmol ½ cystine/mg protein, which is the optimal treatment target. The findings of a more recently published long-term prospective study in a young treatment-naïve cystinosis patient population, corroborate the observations by Langman et al. (Vaisbich et al., JIMD 2022).
Furthermore, the authors of the study by van Stein C. et al. refer to this same observation to argue that the dosing of DR-CYS should be increased from 70-80% to a higher dose. However, we agree with the author of this Letter to the Editor that their proposed total daily dose reduction for DR-CYS to about 70-80% of that of IR-CYS, remains valid, based on the observations of the aforementioned prospective studies (Langman et al., 2012; Langman et al., 2014).

We only do need to point out to the author of this Letter to the Editor that, according to Langman et al. (CJASN 2012), the average total daily steady-state dose of DR-CYS was 82% We agree with the author of this Letter to the Editor that, as demonstrated in the study by Langman et al. (CJASN, 2012), treatment with DR-CYS was non-inferior to IR-CYS, in terms of the fact that the difference observed in the mean peak value of WBC cystine level at 12 hours following administration with DR-CYS was not statistically inferior in comparison to the mean peak WBC cystine value 6 hours following administration with IR-CYS, and that the mean WBC cystine values are below the WBC cystine level target values (< 1 nmol ½ cystine/mg protein) associated with optimal clinical outcomes.
In contrast, the authors of the study described by Van Stein et al. (Orphanet Journal of Rare Diseases, 2021) argue that, based on their observation that following administration of DR-CYS, average WBC cystine levels reach a level above 0.5 nmol cystine/mg protein already after 250min, which is 230min or about 4 hours prior to the next dose of DR-CYS, the dosing interval of DR-CYS should be shortened, and thus dosing frequency of DR-CYS should be increased from twice-daily to three times daily. However, this observation by Stein C. et al is indeed the only basis for changing the recommendation on the dosing frequency of DR-CYS. Indeed, in the 24-months follow-up study by Langman et al. (Journal of Pediatrics, 2014), it was robustly demonstrated that during the entire duration of the study, the average WBC cystine levels remained below the 1 nmol ½ cystine/mg protein, which is the optimal treatment target. The findings of a more recently published long-term prospective study in treatment-naïve patient population in a young cystinosis population, corroborate the observations by Langman et al. (Vaisbich et al., JIMD 2022).
Furthermore, the authors of the study by van Stein C. et al. refer to this same observation to argue that the dosing of DR-CYS should be increased from 70-80% to a higher dose. However, we agree with the author of this Letter to the Editor that their proposed total daily dose reduction for DR-CYS to about 70-80% of that of IR-CYS, remains valid, based on the observations of their aforementioned prospective studies (Langman et al., 2012; Langman et al., 2014).

We only do need to point out to the author of this Letter to the Editor that, according to Langman et al. (CJASN 2012), the average total daily steady-state dose of DR-CYS was 82% of the incoming steady-state total daily dose of IR-CYS, instead of the 91% mentioned here in this manuscript.

**Author Response:**

**Thank you for your thorough assessment. We greatly appreciate the time you dedicated to reviewing this letter and have edited the letter according to your feedback.**

**Original Statement (Paragraph 2, Sentence 5):**

The study met its primary endpoint, with the average steady-state total daily dose of DR-CYS being approximately 91% of the incoming steady-state total daily dose of IR-CYS.^2^

**Revised Statement:**

The study met its primary endpoint, with the average steady-state total daily dose of DR-CYS being approximately 82% of the incoming steady-state total daily dose of IR-CYS.^2^

Sincerely,

Dr. Langman
